# Comparative Analysis of Methods for Assessing On-Target Gene Editing Efficiencies

**DOI:** 10.3390/mps8020023

**Published:** 2025-03-01

**Authors:** Bing Yao, Qiangbing Yang, Manuel A. F. V. Gonçalves, Raymond Schiffelers, Joost P. G. Sluijter, Zhiyong Lei

**Affiliations:** 1 Experimental Cardiology Laboratory, Department of Cardiology, Division of Heart and Lungs, University Medical Center Utrecht, 3508 GA Utrecht, The Netherlands; b.yao@umcutrecht.nl (B.Y.); j.sluijter@umcutrecht.nl (J.P.G.S.); 2 Regenerative Medicine Center Utrecht, Circulatory Health Research Center, University Medical Center Utrecht, University Utrecht, 3584 CT Utrecht, The Netherlands; 3 CDL Research, University Medical Center Utrecht, 3508 GA Utrecht, The Netherlands; 4 Department of Cell and Chemical Biology, Leiden University Medical Centre, 2333 ZA Leiden, The Netherlands

**Keywords:** genome editing, CRISPR/Cas9, editing efficiency, on-target gene editing, genome editing assays

## Abstract

Genome editing based on CRISPR-derived technologies has become a cornerstone in both fundamental research and clinical applications. Accurately measuring editing efficiency is crucial for developing and applying genome editing strategies. This study offers a detailed comparison of widely used techniques for evaluating on-target gene editing efficiency, including T7 Endonuclease I (T7EI), Tracking of Indels by Decomposition (TIDE), Inference of CRISPR Edits (ICE), droplet digital PCR (ddPCR), and live-cell assays involving engineered fluorescent reporter cells. Through a comparative analysis, this study highlights the unique strengths and limitations of each method, aiding researchers in choosing the most appropriate method for their specific needs, ensuring more tailored monitoring of genome editing outcomes in a precise and reliable manner.

## 1. Introduction

Genome editing is a set of promising technologies with the potential to correct genetic diseases [1]. Among the various technologies developed for this purpose, Clustered Regularly Interspaced Short Palindromic Repeats (CRISPR)-based genome editing tools stand out owing to their efficiency and notable versatility [2]. CRISPR-based genome editing technologies, especially those based on well-known CRISPR-Cas9 systems, and advanced platform derivatives, such as cytosine base editors (CBE), adenine base editors (ABE), and prime editors (PE), are revolutionizing our ability to precisely manipulate DNA sequences in cells, plants, and animals [3]. The prototypic CRISPR-Cas9 system, first identified as an adaptive immune system in *Streptococcus pyogenes*, functions to defend the host against invading viruses or foreign plasmids by targeted DNA cleavage [4]. The simplicity and adaptability of this system, which consists of ribonucleoprotein complexes formed by the Cas9 nuclease bound to a CRISPR RNA and a trans-activating CRISPR RNA (crRNA and tracrRNA, respectively), was quickly leveraged to develop molecular tools that can be directed to cut any predefined DNA sequence by engineering single guide RNA (gRNA) molecules containing sequence-specific crRNA and scaffolding tracrRNA moieties [5]. Cas9, directed by the gRNA, induces double-strand DNA breaks (DSBs) at specific locations, after which cellular DNA repair mechanisms, including non-homologous end joining (NHEJ) and homology-directed repair (HDR), are activated to achieve the desired genome editing outcomes [6]. Building on this foundation, further innovations led to the development of the aforementioned CBE, ABE, and PE editors. Base editors, which consist of a nicking Cas9 variant fused to specific nucleotide deaminases, allow for the in situ conversion of DNA base pairs without inducing DSBs, thereby greatly reducing the risk of undesired mutations caused by DSBs [7,8]. PEs, which combine a nicking Cas9 variant with a reverse transcriptase, are guided by a prime editing guide RNA (pegRNA) that also encodes the desired edits. PEs are more versatile than base editors as they facilitate the introduction of well-defined small insertions, deletions, and all of the 12 types of base conversions equally without generating DSBs [9]. Given the specific capabilities and differences among these tools, selecting the appropriate tool for achieving a particular genetic modification goal can be challenging. Therefore, it is crucial to evaluate the efficiency and suitability of each tool to ensure effective and accurate genetic modifications, such as gene corrections.

Various methods have been developed to assess DNA editing efficiencies. The T7 Endonuclease I (T7EI) assay is typically used to detect alleles with small insertions or deletion (indels) caused by the NHEJ-mediated repair of DSBs. The mismatch-sensing T7EI enzyme cleaves heteroduplex DNA fragments created by hybridization between single-stranded PCR products with indel and wildtype sequences, resulting in distinguishable bands on an agarose gel, indicating successful targeted DNA cleavage [10]. Although T7E1 digestion provides quick results, it is only semi-quantitative, even when using densitometric analysis of DNA band intensities (i.e., the ratio of digested to undigested PCR amplicons, representing modified and unmodified alleles, respectively). Hence, T7EI assays lack the sensitivity of more advanced quantitative techniques. Methods such as Tracking of Indels by Decomposition (TIDE) and Inference of CRISPR Edits (ICE) analyze Sanger sequencing chromatograms via sequence trace decomposition algorithms to yield estimations of the frequencies of insertions, deletions, and conversions [11,12,13]. These two Sanger sequencing-based methods offer a more quantitative analysis of gene editing outcomes when compared to those involving T7E1 assays, but their accuracy heavily relies on the quality of PCR amplification and sequencing, which can be a limiting factor under variable experimental conditions or target regions. Droplet digital PCR (ddPCR) offers a different approach, as it measures DNA edit frequencies by using differentially labeled fluorescent probes. Importantly, ddPCR provides for highly precise and quantitative measurements of DNA editing efficiencies and allelic modifications (e.g., NHEJ versus HDR products), which are particularly useful in applications requiring fine discrimination between edit types and the evaluation of edited versus unedited cell frequencies [14]. Finally, fluorescent reporter cells that “light up” upon the installation of specific DNA edits allow for live-cell tracing and quantification of genome editing events via flow cytometry and fluorescence microscopy [15]. Cell-based DNA editing reporter systems are, however, only applicable to cells amenable to engineering and to target sequences placed outside their endogenous chromosomal context. The latter aspect is relevant, as the chromatin environment governing whether DNA sequences are in an epigenetically open or closed configuration has been shown to affect the activity of gene editing tools [16,17].

**Figure 1:**
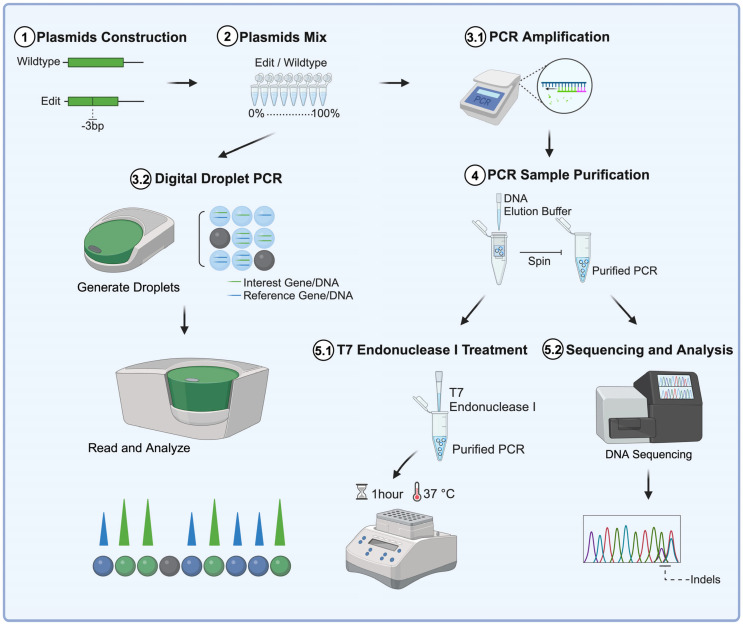
**Comparing DNA editing quantification methods using a plasmid targeting model.** Schematic illustration of the different methods used to assess DNA editing efficiencies in episomal plasmid DNA constructs bearing wildtype and edited sequences.

In gene editing, it is often necessary to screen a large number of gRNAs to identify the one with the highest editing efficiency. Understanding the advantages and limitations of each method allows for a more informed decision-making process in selecting the most appropriate technique(s) for rapid and straightforward screening. A comparative analysis of DNA editing readout methods is therefore essential for testing, applying, and advancing the genome editing field by providing guidance on the selection and optimization of the tools themselves and of the readout methods used for their assessment. In this study, we initiated our evaluation of genome editing frequencies using T7EI, TIDE, ICE, ddPCR, and fluorescent reporter cells by implementing a plasmid target model where constructs with wildtype and edited sequences serve as surrogates of unmodified and modified target alleles, respectively (Figure 1). We compared the advantages and limitations of each method and assessed their performance in terms of reproducibility and versatility. These comparative analyses will help select which method(s) are the most suited for specific genome editing applications.

## 2. Material and Methods

### 2.1. Model Construction

To simulate different levels of editing frequencies, we constructed two plasmids based on the *PLN* R14del and *PLN* WT reporter constructs, each containing a 3-base pair difference. These plasmids were then mixed in varying ratios, ranging from 0% to 100% of each plasmid. The plasmid sequences are provided in Supplementary Table S1.

### 2.2. PCR

The PCR primers are shown in Supplementary Table S2. For PCR reaction, 1 μL of plasmid mixtures, 1 μL of each primer, 10.5 μL of RNase-free water, and 12.5 μL of Q5 Hot Start High-Fidelity 2X Master Mix (M0494, New England Biolabs, Ipswich, MA, USA) were used in a final reaction volume of 25 μL performed in a C1000 Touch thermal cycler (BioRad Laboratories, Hercules, CA, USA). The PCR thermocycling program used was as follows: initial denaturation step at 98 °C for 30 s, followed by 30 cycles of denaturation at 98 °C for 10 s, annealing at 60 °C for 30 s, and extension at 72 °C for 30 s. A final extension step was carried out at 72 °C for 2 min. The PCR products were run on a 1% agarose gel containing Ethidium Bromide Solution (21-1056334, Invitrogen, Waltham, MA, USA) and imaged in the ChemiDoc MP imaging system (BioRad Laboratories, Hercules, CA, USA). Sanger sequencing was performed by Macrogen.

### 2.3. Determining Gene Editing Frequencies Using T7 Endonuclease I

The PCR products were purified by the Gel and PCR Clean-Up Kit (2104/001, Macherey-Nagel, Düren, Germany). For T7 treatment, 8 μL of purified PCR products, 1 μL of NEBuffer2, and 1 μL T7 Endonuclease I (M0302, New England Biolabs, Ipswich, MA, USA) were incubated at 37 °C for 30 min. The T7E1-treated samples were run on a 1% agarose gel with Ethidium Bromide Solution (21-1056334, Invitrogen, Waltham, MA, USA) or GelRed Nucleic Acid Stain 10,000× Water (41003-T, Biotium, Fremont, CA, USA). Both stains were used at a 1:10,000 dilution, and the gels were imaged in the ChemiDoc MP imaging system (BioRad Laboratories, Hercules, CA, USA). The ratio of uncleaved bands and cleaved bands was analyzed by Imaging Lab.

### 2.4. Determining Gene Editing Frequencies Using TIDE

Sanger sequencing chromatograms were obtained from all the edited and wildtype samples in the *.ab1 format, ensuring that the target site-specific sequencing was successful in all the samples. The TIDE analysis website (http://shinyapps.datacurators.nl/tide/, accessed between 3 September 2021 and 30 July 2025) was used to analyze the sequencing data.

First, the wildtype (non-edited) and the edited sample sequencing files (.ab1) were uploaded. The TIDE tool uses the wildtype sequence as a reference to detect any insertions or deletions (indels) introduced at the target site in the edited sample. The exact sequence position where the CRISPR/Cas9 cut site was located, being usually 3 bases upstream of the PAM sequence, was used as input. A window of base pairs around the cut site was selected for analysis. A typical window of 100–200 bps was chosen, which is sufficient to capture most of the editing events while avoiding noise from the sequence ends. The window started 50–100 bps before the cut site and extended 50–100 bps after it to ensure potential indels and any sequencing variability near the ends of the reads were considered. While the default parameters are generally sufficient, the “indel range” parameter was adjusted in this experiment to detect indels of varying sizes. The alignment window was set from 500 to 539, while the decomposition window ranged from 557 to 620. An indel size of 3 base pairs was used for our analysis. The TIDE tool aligned the edited chromatogram with the wildtype chromatogram and decomposed the mixture of sequences into a proportion of wildtype and indel-containing alleles. TIDE identified the most common indels based on the differences in the signal intensity at each base. The output provided the percentage of alleles containing indels compared to wildtype alleles, the sizes of the most frequent indels, and their corresponding frequencies within the sample.

### 2.5. Determining Gene Editing Frequencies Using ICE

The ICE analysis website (https://ice.synthego.com/#/, accessed between 3 September 2021 and 30 July 2025) was used to analyze the sequencing data. For the analysis, the Sanger sequencing file (*.ab1) was uploaded for the non-edited (wildtype) sample and all the edited samples. ICE compares the chromatograms of the edited sample against the wildtype reference to identify insertions or deletions (indels). An input gRNA sequence was used to detect editing, which is essential for locating the target site and identifying indels near the cleavage site. ICE automatically identifies the most likely CRISPR cut site, and default settings typically provide accurate results. ICE aligns the sequencing chromatograms from the edited and wildtype samples, decomposing the mixture into wildtype and edited alleles, and calculates both the editing efficiency and the indel distribution. Additionally, ICE provides a Q-score, which represents the quality of the Sanger sequencing data. A higher Q-score indicates better sequencing quality.

### 2.6. Droplet Digital PCR and Data Analysis

For ddPCR, specific primer pairs and hybridization probes for the wildtype and the mutant assays were designed (Supplementary Table S3). First, ddPCR 0.1 pg plasmid DNA, a 0.5 μL 40× Probe assay, and 11 μL ddPCR Supermix for Probes (No dUTP) (186-3023, BioRad Laboratories, Hercules, CA, USA) were added and supplemented with nuclease- and protease-free water to a final volume of 22 μL. Droplets were generated using the QX200 Automated droplet generator (BioRad, Feldkirchen, Germany) according to the manufacturer’s instructions. PCR amplifications were performed in a thermal cycler (BioRad, Feldkirchen, Germany) with the following conditions: 98 °C for 1 min; 40 cycles of 98 °C for 10 s, 57.5 °C for 30 s, and 72 °C for 60 s, followed by 72 °C for 5 min. After ddPCR, the samples were measured and analyzed by the QX200 Droplet Reader (BioRad, Feldkirchen, Germany).

### 2.7. Cell Culture and Transfection

The HEK293T CRISPR-Cas9-activated fluorescence reporter cells were cultured in Dulbecco’s Modified Eagle Medium (DMEM, 10564011, Thermo Fisher Scientific, Waltham, MA, USA) with 100 µg/mL streptomycin, 100 U/mL penicillin (15140122, Thermo Fisher Scientific, Waltham, MA, USA), and 10% fetal bovine serum (FBS, MFCD00132239, Sigma-Aldrich, Germany) at 37 °C and 5% CO_2_. The transfection experiments were performed in 12-well plates. We transfected the cell at a density of 60% after 24 h of seeding using Lipofectamine 3000 Reagent (#L3000001, Thermo Fisher Scientific, Waltham, MA, USA). A gradient of total DNA amounts of 100 ng to 1000 ng was used in the transfection at a ratio 1:9 of Cas9:sgRNA. After 72 h, the cells were detached and centrifuged for 5 min at 300× *g* and then resuspended by 200 μL PBS. The cells were analyzed on an Invitrogen EVOS FL Digital Inverted Fluorescence Microscope (Thermo Fisher Scientific, Waltham, MA, USA) and a CytoFlex Flow Cyto (Beckman Coulter Life Sciences, Brea, CA, USA). The protospacer sequence of the guide RNA used in this study is as follows: 5′-GGACAGTACTCCGCTCGAGT-3′.

## 3. Results

### 3.1. Assessing Gene Editing Frequencies by Using T7 Endonuclease I Assays

To evaluate and compare DNA editing frequencies in a controlled manner, we generated a series of surrogate samples. In particular, we mixed plasmids containing sequences derived from the wildtype phospholamban gene (*PLN* WT) and from the same phospholamban gene with a three-base pair deletion corresponding to the arginine 14 codon (*PLN* R14del). The former and latter sequences represent unmodified and edited *PLN* alleles, respectively. These two different alleles were mixed in proportions ranging from 0% to 100%. Typically, in the context of in cellulo genome editing experiments, genomic DNA samples contain (i) indels with varying sizes resulting from NHEJ-mediated repair of site-specific DSBs or (ii) well-defined edits derived from precise HDR-mediated repair of site-specific DSBs with tailored donor DNA templates.

**Figure 2:**
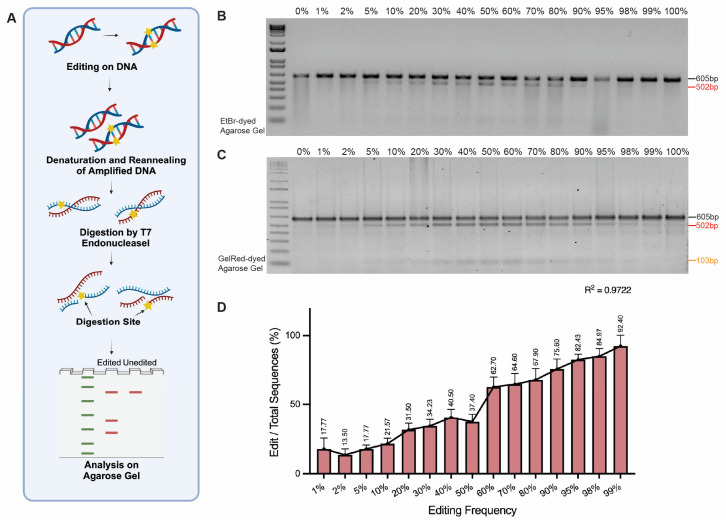
Analysis of genome editing using the T7 Endonuclease I (T7EI) assay. (**A**) Schematic representation of the T7EI assay. Target DNA is subjected to PCR amplification with the products being then denatured and reannealed via a defined thermocycling program (provided in Materials and Methods). Heteroduplexes containing nucleotide mismatches are selectively digested by T7EI, generating DNA fragments that are analyzed through agarose gel electrophoreses and, if needed, densitometry measurements (not shown). Typically, in the context of in cellulo genome editing experiments, the nucleotide mismatches correspond to indels with varying sizes resulting from NHEJ-mediated repair of site-specific DSBs or to well-defined edits derived from precise HDR-mediated repair of site-specific DSBs with tailored donor DNA templates. (**B**,**C**) Agarose gel with undigested and T7EI-digested DNA species stained with EtBr and GelRed, respectively. The 605 bp fragments (black arrows) indicate undigested PCR products. The 502 bp and 103 bp fragments (red and yellow arrows, respectively) indicate the DNA fragments resulting from T7EI-induced DNA cleavage at the mismatched site. Note: The detection of the 103 bp DNA species is substantially more discernible when using the GelRed dye. (**D**) The bar chart represents the percentage of edit bands relative to the total DNA bands observed at various editing frequencies (0–100%). The data are represented as the mean ± SD from biological replications (n = 3). The R^2^ value demonstrates the correlation between the theoretical editing frequencies and the observed editing frequencies.

The T7EI enzyme has the ability to specifically bind to and cleave at sites with base pair mismatches regardless of whether these mismatches are caused by single or multiple nucleotide differences amongst the two DNA chains [10,18]. The T7E1 enzyme recognizes and cleaves distorted DNA structures, called kinks, caused by mismatched nucleotides, making it suitable for detecting multiple DNA edits spanning from single to multiple base pairs (bps) [19] (Figure 2A). Hence, our 3 bp *PLN* codon deletion edit model is expected to be compatible with T7EI assays for assessing DNA editing frequencies. Indeed, after treating PCR products spanning the 3 bp polymorphism with T7EI, agarose gel electrophoresis revealed the presence of two or three distinct bands, which are diagnostic for DNA edit detection (Figure 2B). We observed that DNA samples with 5% to 90% of the 3 bp deletion template consistently exhibited two bands, suggesting that the T7EI assay is most sensitive within this range of biallelic mixtures. To enhance the detection of T7EI digestion products, we used the GelRed stain instead of the ethidium bromide (EtBr) DNA intercalator due to its superior sensitivity and safety profiles [20]. This switch enabled the detection of a broader range of biallelic mixtures (i.e., 1% to 98% edits), thereby significantly improving the assay’s sensitivity (Figure 2C). However, the analyzed result showed that the assay performs best within the 40% to 60% editing range (Figure 2D). Furthermore, the sensitivity of GelRed staining allowed for the detection of smaller cleavage fragments, such as the 103 bp band, which were not observed on EtBr-dyed gels. Although these smaller fragments were not consistently detected under all conditions, this may be due to a lower indel frequency, where the resulting cleavage fragments are present in smaller quantities and are difficult to detect because of limitations in gel resolution or staining sensitivity. This improvement in fragment detection increases the effectiveness of T7E1 assays, providing a more cost-efficient, rapid, and straightforward method for evaluating DNA samples.

### 3.2. Assessing Gene Editing Frequencies by Using TIDE Analysis

Sanger sequencing-based methods are commonly used to determine the performance of gene editing tools because of their ability to provide information on edit frequencies together with edit type and size distributions. Among these, TIDE is a user-friendly method, requiring only the control and experimental sequence traces retrieved from mock- and gene-edited cells, respectively, alongside the guide RNA sequence [13,21]. This method involves establishing the expected DNA breakage site positions and subsequently determining edit frequencies by trace sequence decomposition after comparing control and experimental sample chromatograms [11]. Notably, the TIDE algorithm can detect edit frequencies as low as 2.5%; however, the accuracy of this method is heavily dependent on the quality of the PCR amplicons and Sanger sequencing reads [11].

**Figure 3:**
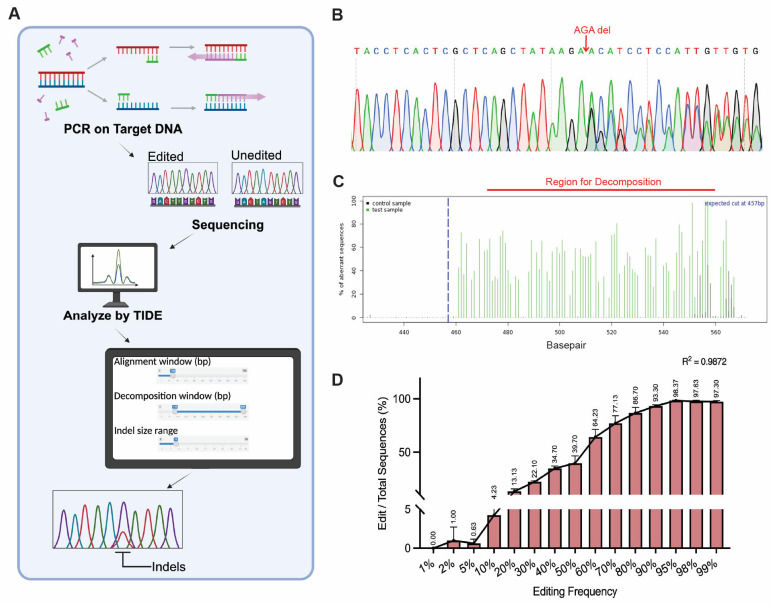
**Analysis of genome editing frequencies using Tracking of Indels by Decomposition (TIDE) assay.** (**A**) Schematic representation of the TIDE workflow. Target DNA is subjected to PCR followed by Sanger sequencing of products derived from edited and wildtype sequences. The resulting chromatograms are then analyzed to estimate the edit frequencies. Parameters such as the alignment window, decomposition window, and indel size range are set for analysis. (**B**) Representative Sanger sequencing chromatogram showing a 3 bp deletion (AGA) in the edited sequence compared to the wildtype. The region with the edit is highlighted. (**C**) TIDE analysis quality control plot showing the percentage of the aberrant sequence signal. The blue dashed line marks the expected cut site, while the region for decomposition is indicated in red, showing the region from where the software calculates the editing frequency. (**D**) TIDE analysis of DNA samples containing defined ratios between edited and wildtype sequences. The edit rates were determined by TIDE analysis; the frequencies of the edited sequences are plotted. The data are represented as the mean ± SD from biological replications (n = 3). The R^2^ value demonstrates the correlation between the theoretical editing frequencies and the observed editing frequencies.

In our study, we applied TIDE for analyzing DNA samples containing defined ratios between edited and wildtype sequences spanning from 0% to 100% (Figure 3A). The Sanger sequencing results showed overlapping peaks. For instance, in the sample containing a 50% mix of edited and wildtype DNA, double peaks were observed at several positions (Figure 3B). Given appropriate settings for alignment, decomposition windows, and indel sizes (Figure 3C, details in Methods and Materials), TIDE successfully detected a 3 bp deletion edit at frequencies ranging from 2% to 99%. However, the TIDE analysis demonstrated limitations in accuracy when the editing frequencies were below 20% or above 90%, particularly underestimating the editing levels when the mutant plasmid was below 50% and overestimating them when it exceeded 60% (Figure 3D). These observations suggest that TIDE is most robust for samples with moderate-to-high editing levels. Therefore, for experiments anticipating low editing frequencies (<10%), TIDE may not provide reliable results.

### 3.3. Assessing Gene Editing Frequencies by Using ICE Analysis

**Figure 4:**
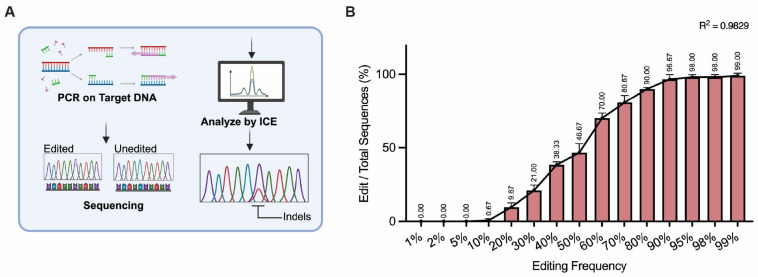
Analysis of genome editing frequencies by using Inference of CRISPR Edits (ICE). (**A**) Schematic overview of the ICE workflow. Target DNA is subjected to PCR followed by Sanger sequencing of products derived from edited and wildtype sequences. The resulting chromatograms are then analyzed to estimate edit frequencies using the ICE software. (**B**) ICE analysis of DNA samples containing defined ratios between edited and wildtype sequences. The edit rates were determined by ICE analysis; the frequencies of the edited sequences are plotted. The data are represented as the mean ± SD from biological replications (n = 3). The R^2^ value demonstrates the correlation between the theoretical editing frequencies and the observed editing frequencies.

The ICE algorithm is another method that, like TIDE, allows for assessing edit frequencies together with edit type and size distributions by using Sanger sequencing data as input. However, when compared to TIDE, the ICE algorithm offers the advantage of automated analysis without the requirement of manual parameter adjustments (Figure 4A) [12]. In our study, when analyzing the same Sanger sequencing data with ICE, the minimum detectable editing frequency was found to be 20%. ICE underestimated editing levels in samples where the mutated plasmid constituted less than 50% of the sample and overestimated editing levels in samples where it exceeded 60% (Figure 4B). Notably, significant discrepancies were observed between the measured and theoretical values at editing frequencies below 30% or beyond 70%, indicating reduced accuracy in these ranges.

### 3.4. Assessing Gene Editing Frequencies by Using ddPCR

**Figure 5:**
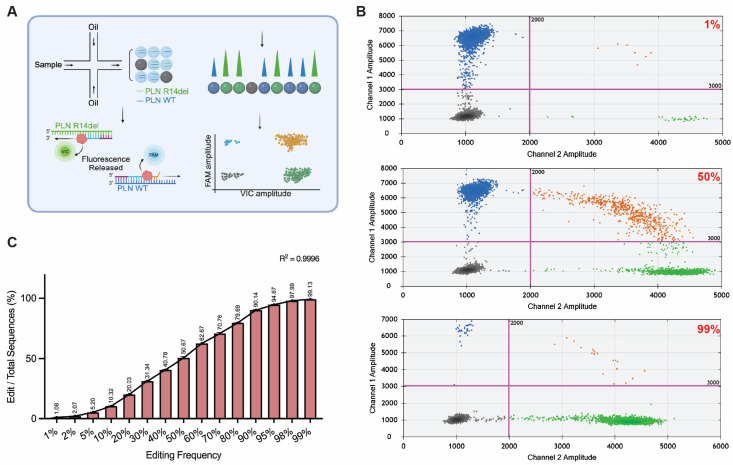
**Analysis of genome editing frequencies by using droplet digital PCR (ddPCR).** (**A**) Schematic illustration of the digital droplet PCR method tailored for determining genome editing frequencies. The PCR sample is partitioned into thousands of oil droplets, each containing either the edit or reference wildtype sequences. Fluorescent probes bind specifically to the edit (FAM) and reference (VIC) sequences, releasing fluorescence upon amplification. The resulting fluorescence amplitudes are measured and analyzed to quantify the proportions of edited sequences in each sample. (**B**) Representative scatter plots of droplet fluorescence amplitudes at different editing frequencies (1%, 50%, and 99%). The *x*-axis represents FAM fluorescence (Wildtype allele), and the *y*-axis represents VIC fluorescence (mutant allele). Each dot corresponds to a droplet, and the colors represent wildtype droplets (blue), mutant droplets (green), double-positive droplets (orange), and negative droplets (gray). (**C**) ddPCR analysis of DNA samples containing defined ratios between edited and wildtype sequences. The edit rates were determined by ddPCR; the frequencies of the edited sequences are plotted. The data are represented as the mean ± SD from biological replications (n = 3). The R^2^ value demonstrates the correlation between the theoretical editing frequencies and the observed editing frequencies.

ddPCR combines PCR with fluorescent probes to form a remarkably accurate and sensitive method that is becoming more extensively utilized in both clinical diagnostics and research [11,12,22]. In ddPCR, a DNA sample mixed with PCR reagents, target-specific primers, and fluorescent probes is subdivided into thousands of nanoliter-sized droplets, with each droplet serving as an independent PCR reaction chamber. During PCR amplification, the presence of target DNA in a droplet induces fluorescence, which is then quantified by a droplet reader to distinguish droplets containing target DNA. This allows for the absolute quantification of target DNA, offering exceptional accuracy, including for the detection of different types of genome editing events in a quantitative manner. Specifically, ddPCR employs differently labeled fluorescent probes that specifically bind to either the wildtype or edited allele and reference alleles (Figure 5A) [23]. Typically, housekeeping genes serve as reference sequences in cellular models to normalize for variations in DNA input and PCR efficiency. However, as our plasmid-based DNA editing model lacks housekeeping genes as such, we used a wildtype gene probe as the internal control. This adaptation ensures accurate normalization of total DNA input and PCR efficiency across DNA samples and experiments. We employed probes tagged with FAM and VIC fluorescent dyes to target the wildtype and edit-containing sequences, respectively (Figure 5B). The results demonstrated that ddPCR is capable of detecting DNA editing frequencies ranging from 1% to 99% with exceptional effectiveness (Figure 5C).

### 3.5. Assessing Gene Editing Frequencies by Using Reporter Cells

**Figure 6:**
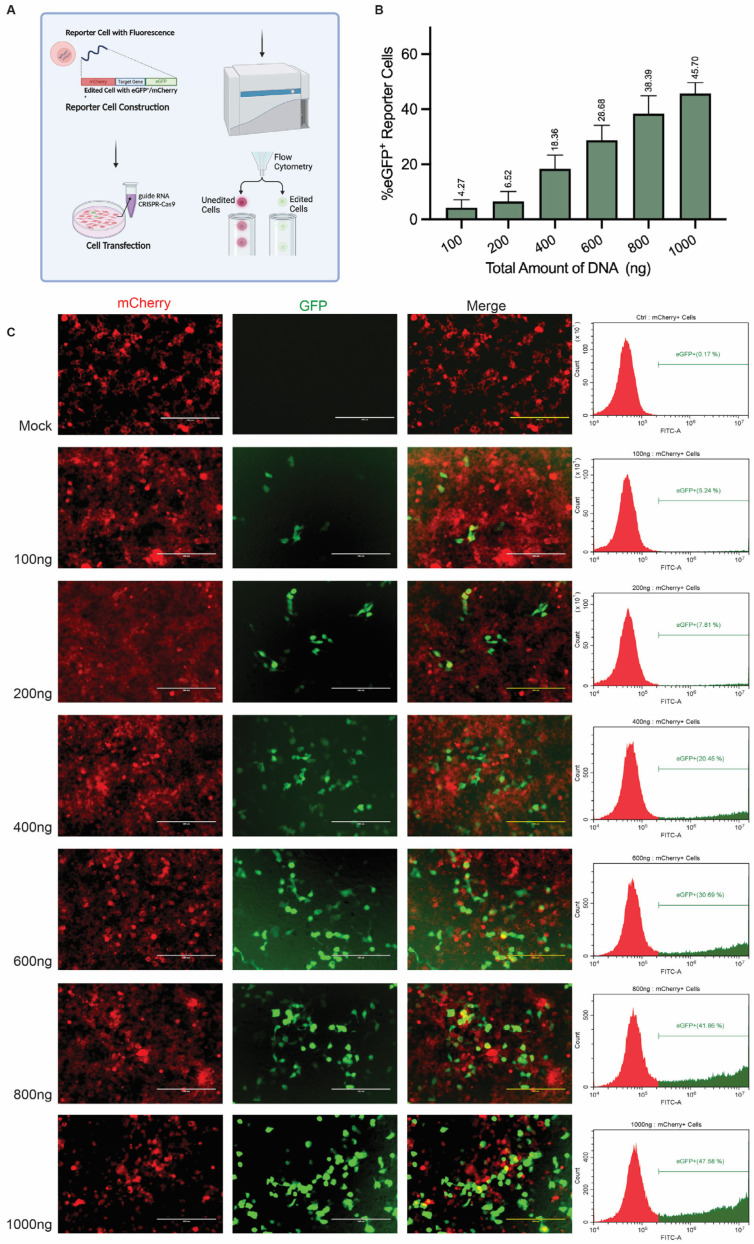
**Analysis of genome editing efficiencies using reporter cells.** (**A**) Schematic representation of the CRISPR-Cas9-activated fluorescence reporter cell system and experimental workflow for evaluating gene editing frequencies. (**B**) Flow cytometry analysis showing the percentage of eGFP^+^ reporter cells upon treatment with different amounts of plasmid DNA encoding SpCas9 and an eGFP-specific gRNA (100–1000 ng). The data are represented as the mean ± SD from biological replications (n = 3). (**C**) Fluorescent microscopy images of stable CRISPR-Cas9-activated fluorescence reporter cells after the transfection of SpCas9 and gRNA plasmids (edited cells express eGFP). The scale bar represents 200 µm. Representative images as observed after three independent experiments.

Live-cell reporter cell lines are valuable methods for detecting and quantifying gene editing events involving reporter gene knockout or frameshift-mediated repair induced by treatments with specific gene editing reagents [24]. We employed the HEK293T CRISPR-Cas9-activated fluorescence reporter cell line, which contains a “Stoplight” reporter system (Figure 6A) [15]. In this system, a reporter becomes permanently activated following Cas9:gRNA-induced DSB formation at a specific sequence upstream of a frame-shifted eGFP gene. NHEJ-mediated DSB repair and the ensuing installation of indels leads to reading frame resetting in a fraction of the treated cells with the resulting transgene expression serving as a surrogate for the activity of the CRISPR-Cas9 nuclease complex used. To assess this method, we co-transfected the reporter cells with varying amounts of plasmids encoding Cas9 and an eGFP-targeting gRNA, ranging from 100 ng to 1000 ng. Subsequently, we employed flow cytoflex to quantify the eGFP-positive cells, each of which represents individual gene editing events. The gene editing frequencies varied in a dose-dependent manner from 4.27% to 45.70%, showing a fairly broad detection range (Figure 6B,C).

## 4. Discussion

In this study, we performed a systematic comparative analysis of T7EI, TIDE, ICE, ddPCR, and reporter cell assays to assess their performances in detecting and quantifying DNA editing events. We summarize the resulting findings in Table 1. Although ddPCR and reporter cells were used to assess different types of edits, ddPCR accurately measured the 3 bp deletion in gene editing, while reporter cells provided a qualitative proxy for detecting gene edits mediated by NHEJ, as indicated by eGFP expression. While eGFP expression serves as an indirect readout, it does not directly quantify editing frequencies. Therefore, the claim of high accuracy and sensitivity in measuring and detecting DNA editing frequencies should be interpreted in the context of functional detection rather than precise quantification. Their performance is in fact superior to that of the other three methods, i.e., T7EI, TIDE, and ICE. T7EI assays based on GelRed staining seem particularly useful for obtaining quick results regarding the relative activity of different CRISPR-Cas9 complexes, making them ideal for initial screenings where time and cost constraints are important factors. While ICE is highly effective and accurate for projects where high levels of editing are anticipated, its sensitivity is significantly reduced when editing efficiency is low, with a minimum detectable editing frequency of 20%. TIDE, by contrast, demonstrates a greater ability to detect low-frequency edits, with a minimum detectable editing frequency of 10%. However, TIDE tends to underestimate the true level of editing, particularly in samples with editing frequencies below 50%. Although neither method can precisely quantify the exact level of editing, TIDE appears to outperform ICE in detecting edits at lower frequencies. Researchers should carefully evaluate these strengths and limitations when choosing the most suitable method for their specific experimental requirements.

Clearly, each method also has its own set of limitations. For instance, T7EI assays, while offering quick and economical detection of gene editing events, they do not yield sequence-specific information on the types and locations of DNA changes. In contrast, TIDE and ICE assays offer effective edit quantification as well as indel type and location identification, but can be constrained by the quality of input sequence traces and the somewhat higher costs associated with downstream Sanger sequencing. ddPCR methodologies require specific fluorescent probes and equipment but deliver high sensitivity and accuracy while being also amenable for analyzing large sample sizes. Finally, readouts based on reporter cells, although accurate and sensitive, require the establishment of specific reporter cell lines for each target sequence of interest, making them a time-consuming assay with limited versatility.

Limitation of this study: This study is based on the 3bp deletion plasmid model; therefore, it is highly standardized and homogeneous. In real experiments, where the samples could be more complicated, the instructions provided by this study need to be verified.

In conclusion, each method possesses unique advantages and limitations for assessing gene editing activities and outcomes. The choice of a particular method should be contingent upon the specific needs and throughput of the research project at hand and consider factors such as the type of desired information, the DNA sample quality and quantity, and the availability of resources and equipment. Finally, employing a combination of methods should, clearly, provide for a more comprehensive and accurate evaluation of gene editing frequencies and outcomes.

## Figures and Tables

**Table 1:** Summary of the different features related to the indicated DNA editing detection methods.

Method	Detection Range (%)	Accuracy	Time Expenditure	Costs	Specific Requirements	Applications
T7EI	1–98	Low	Low	Low	T7EI enzyme and electrophoreses apparatus	[25,26,27,28]
TIDE	2–99	Medium	Medium	Medium	High-quality sequence reads	[29,30]
ICE	20–99	Low	Medium	Medium	High-quality sequence reads	[31,32,33]
ddPCR	0–99	High	Low	High	Probe assay, droplet generator, and droplet reader	[34,35]
Reporter cell line	0–100	High	High	High	Fluorescent reporter cell line establishment, and flow cytometer	[36,37,38]

## Data Availability

No publicly archived datasets were analyzed or generated during the study.

## Supplementary Materials

The following supporting information can be downloaded at: https://www.mdpi.com/article/10.3390/mps8020023/s1, Table S1: Plasmid sequence; Table S2: Primers and probes for PCR; Table S3: Primers and Probes for ddPCR.
